# Android Application Model of “Suami Siaga Plus” as an Innovation in Birth Preparedness and Complication Readiness (BP/CR) Intervention 

**Published:** 2017-03

**Authors:** Hanna Yuanita Dana Santoso, Supriyana Supriyana, Bahiyatun Bahiyatun, Melyana Nurul Widyawati, Diyah Fatmasari, Sudiyono Sudiyono, Dyah Anantalia Widyastari, Doni Marisi Sinaga

**Affiliations:** 1 Postgraduate Midwifery Program, Poltekkes Kemenkes Semarang , Semarang, Indonesia; Akademi Kebidanan Panti Wilasa, Semarang, Indonesia; 2 Postgraduate Midwifery Program, Poltekkes Kemenkes Semarang, Kota Semarang, Indonesia; 3 Institute for Population and Social Research, Mahidol University, Bangkok, Thailand; Yayasan Aliansi Cendekiawan Indonesia Thailand (Indonesian Scholars' Alliance), Semarang, Indonesia; 4 International Program in Hazardous Substance and Environmental Management, Graduate School, Chulalongkorn University, Bangkok, Thailand; Yayasan Aliansi Cendekiawan Indonesia Thailand (Indonesian Scholars' Alliance), Semarang, Indonesia

**Keywords:** Birth Preparedness and Complication Readiness (BP/CR) Score, Alert Husband, Technology in Birth, Suami Siaga Plus Android Application, Mobile Health (M-Health)

## Abstract

**Objective:** WHO recommends *Mobile health*, a practice of medicine and public *health* supported by *mobile* devices, to improve community health status and change people's behavior for the health purposes. The present study sought to examine the effectiveness of the android application program of *Suami Siaga Plus* in increasing husband’s scores in birth preparedness and complication readiness (BP/CR) intervention.

**Materials and methods:** It was a randomized controlled trial with pretest-posttest design. A total of 38 couples of husbands and pregnant women from three health centers at three sub districts in Semarang was selected by proportional systematic random sampling technique and equally distributed into control and intervention group. A questionnaire related to BP/CR published by JHPIEGO was employed in data collection. Statistical analysis was performed to obtain the frequency distribution and percentage of the variables, and also to assess the mean difference of BP/CR score of husbands.

**Results:** Husbands’ knowledge of key danger signs and five standard elements in BP/CR practices in both intervention and control group increased after counseling. Moreover, the proportion of husbands who understand the key danger signs during pregnancy was higher among those who were exposed by *Suami Siaga Plus* application delivered via mobile phone. Counseling only increased husbands’ score from 61.5 to 62.6 (2%), whilst the combination of counseling and the application boosted 20% of husbands’ score from 60.4 to 72.9 (p-value 0.000).

**Conclusion:** A combination of counseling and *Suami Siaga Plus *application significantly improves husbands and wives’ score on BP/CR compared to those who received counseling only. The data suggests the application would be able to suppress the three delays, which in turn can reduce the maternal mortality rate. The study results could be important information for the Department of Health and health professionals to use android application program*,* in particular to the husband whose wife is in pregnancy, childbirth and postpartum periods.

## Introduction

Maternal death is one of important indicators of a country development. Indonesia targeted MM Ratio at 102 per 100,000 live births in 2015 by implementing programs to reduce maternal deaths. Indonesian Demographic and Health Survey recorded, Indonesia have succeed in decreasing MM Ratio from 390 deaths per 100,000 live births in 1997 into 307 and 228 in 2002 and 2007, respectively. Nevertheless, Indonesia was failed in maintaining its achievement because MM Ratio was elevated to 359 per 100,000 live birth (1-3). 

Various efforts in reducing maternal deaths have been implemented, including using medical technology in order to lessen the three delays which identified as the main cause of deaths(4). Community empowerments also have involved husbands as the key decision makers in the family especially in fertility and health-related decision including pregnancy, childbirth and postpartum. Studies found, husband involvement is one of the key factors in reducing maternal deaths (5, 6). Nevertheless, husband could also be a barrier to maternal health especially when he is not prepared for complications, has fear and confusion and ultimately unable to make an important decision immediately. Ideally, husband should be able to take the best decision for the health of mother and child, and the best decision is supported by the preparedness to face obstetric complications (7-10).

Husband empowerment program in Indonesia was proclaimed and embodied in the form of *a national alert husband* campaign in 1996, as a support to family planning program and population policy at the end of Suharto era. The policy to involve men in the past was born to respond the social development where women and men should bear an equal position in the household (11). The notion of ALERT/SIAGA itself is an abbreviation of Ready (*SIap*), Take (*Antar*) and Stand by (*jaGA*). Although it has been long established, studies found that many husbands were limited to only understand this as an invitation to accompany their wife, and had less attention to birth preparedness and complication readiness (12).

Related to the family empowerment, in 2014, Johns Hopkins Program for International Education in Gynecology and Obstetrics (JHPIEGO) published a set of instruments to assess the preparation for childbirth and preparedness for obstetric complication called Birth Preparedness and Complication Readiness (BP/CR) toolkit. BP/CR is a strategy to promote optimal care, especially preparation for labor and delivery and ready to face the obstetrics complications with the aim of reducing the three delays. BP/CR intervention has been widely promoted at various international institutions to reduce maternal health risks (13). 

Accompanying the instrument’s development, technology has been used widely to expand its coverage. With the penetration of internet at individual basis such as smartphones, mobile health (M-Health) becomes one of WHO recommendations as a breakthrough in health care. Given the nature of mobile phones, health information can be provided in a more effective and efficient way since the users can retrieve the information anytime at their convenience (14). The anonymity, affordability and availability of media have indeed directing health program into a new stage where technology can help to solve healthcare challenges by reducing health costs and waiting time for treatment, and also to improving access to health care. M-Health supports health programs through referrals and searches, decision making support, supervision, scheduling and tracking the follow up visits, as well as health education and counseling. Previous studies found, health programs conducted based on mobile devices are seen as a more cost and energy effective approach compared to manual program (e.g., counseling, leaflets, and brochures) (15-17). The present study sought to examine the effectiveness of the android application program of *Suami Siaga Plus *in increasing husband’s scores in birth preparedness and complication readiness intervention.

## Materials and methods

The study was a randomized controlled trial with pretest-posttest design. Participants were assigned in intervention or control group using closed envelopes. A total of 38 couples of husband and pregnant women from three health centers at three sub-districts in Semarang named Bandarharjo, Karangdoro and Bugangan was selected by proportional systematic random sampling technique and equally distributed into control and intervention group. Couples were eligible for randomization if the wife was in the third trimester of pregnancy during the study period. All patients signed informed consent form prior to their participation.

Couples from both groups received counseling, but only husbands from the intervention group installed an android application called *Suami Siaga Plus*. There is no restriction for the wife to access the program. The application program provided 4 main features included information on (1) woman’s characteristics (age, parity, Hb levels, height, birth space and upper arm circumference) and three gestational stages comprised of (2) pregnancy; (3) childbirth; and (4) postpartum. Under pregnancy features, the application helps to record the first day of last menstrual period to calculate the gestation age, women’s complaints during pregnancy, and key danger signs and complication during pregnancy stage. In addition, the application also provides a reminder for ANC schedule, completed with the information of fetus growth and development and also pregnancy exercise for pregnant women. On childbirth features, husbands will be directed to understand the key danger signs during delivery, and will be requested to record the wives’ complaints (if any) during partum. *Suami Siaga Plus* also covers post-partum period where husbands will be notified of the key danger signs during neonatal, also identifying other key danger signs by paying attention to wives’ complaints. Users will also be notified of care and treatment that should be given for post-partum women on specific period such as 0-24 hours, 1-7 days, 1-2 weeks and 3-6 weeks after delivery. Child care, breastfeeding technique, and post-partum exercise are also provided under childbirth features.

A questionnaire related to the Birth Preparedness and Complication Readiness (BP/CR) toolkit published by JHPIEGO was distributed to the couples during their 3-time visit to the health center. The first visit was intended to measure the baseline BP/CR score (pretest) in the third trimester of pregnancy, before the application installed. The next follow-up was conducted 3 weeks after the first visit to measure the BP/CR posttest score. The last encounter was conducted in postpartum period to measure the Complication Readiness score during postpartum. Statistical analysis was done to show the frequency distribution and percentage of the variables. Later, dependent and independent T-tests were employed to measure the mean difference of BP/CR scores of husbands who were involved in *Suami Siaga Plus *program as a tool in birth preparedness and complication readiness. 

The present study obtained its approval from Ethical Committee Board of Politeknik Kesehatan Kemenkes Semarang (*Semarang Health Polytechnic)*, Semarang, Indonesia, with reference number 016/KEPK/Poltekkes-Smg/EC/2017, on January 9^th^, 2017.

## Results

***Respondents’ characteristics:*** Husbands as the main subjects of the study aged between 21 to 45 years old, with the mean age at 32 years. All of them were fully employed although their education varied; mostly completed secondary (81%) and only few of them attained primary (13.2%) and higher education (5%). The wives are relatively younger, aged between 17 to 41 years old, with mean age of 28.9 years. Most wives were unemployed (34%) and completed only secondary level of education (65%). The vast majority of the women were primigravida (52%), but some of them were experiencing their second (13%) or third pregnancy (7%).

***Husbands’ BP/CR score description:*** Table 1 shows that husbands ‘knowledge of key danger signs and five standard elements in BP/CR practices in both intervention and control group increased after counseling. 

**Table 1:** BP/CR score among Husbands

**Indicators**	**Intervention**		**Control**	
	**Before**	**After**	**Before**	**After**
Knowledge of key danger signs				
During pregnancy	7 (36.8%)	16 (84.2%)	7 (36.8%)	10 (52.6%)
During labor	6 (31.6%)	15 (78.9%)	7 (36.8%)	8 (42.1%)
During postpartum	6 (31.6%)	15 (78.9%)	6 (31.6%)	9 (47.4%)
Five standard elements in BP/CR practices				
Choose an appropriate birth location	18 (94.7%)	18 (94.7%)	17 (89.5%)	17 (89.5%)
Give birth with a skilled provider	17 (89.5%)	19 (100%)	17 (89.5%)	19 (100%)
Mode of transport	9 (47.4%)	19 (100%)	14 (73.7%)	16 (84.2%)
Blood donor in emergency case	5 (26.3%)	7 (36.8%)	7 (36.8%)	7 (36.8%)
Save money	15 (78.9%)	17 (89.5%)	15 (78.9%)	16 (84.2%)

**Table 2:** The total BP/CR score before and after intervention among men

**Total BP/CR Score**		**Group**		**p value**
		**Android App and Counseling**	**Counseling**	
1	Before intervention			0.718
	a. Mean ± SD	60.4 ± 8.7	61.5 ± 8.9	
	b. Min-max	40.8-76.1	49.3-74.6	
2	After intervention: Follow up 1			0.001
	a. Mean ±SD	72.9 ± 9.0	62.6 ± 8.9	
	b. Min-max	43.7-85.9	49.3-76.1	
3	After intervention: Follow up 2			0.000
	a. Mean ±SD	81.8 ± 6.2	72.3 ± 7.9	
	b. Min-max	66.7-88.1	54.8-83.3	

Moreover, the proportion of husbands who understand the key danger signs during pregnancy was higher among those who were exposed by *Suami Siaga Plus* application delivered via mobile phone. From the BP/CR practices, the data suggests the program installed on the mobile phone will determine decision to choose mode of transport to send their wife to the nearby hospital. The results also noted that only few husbands declared their decision to save money and willing to donor their bloods in emergency cases after obtained counseling and received information from the application. Table 1 also shows husbands’ decision to choose appropriate location for birth was not defined by the counseling, even combined with *Suami Siaga Plus *installment in their mobile phone.

Table 2 indicates the total BP/CR score of the male participants was varied ranged 40.8 to 76.1. There was no difference in husbands’ BP/CR scores in both intervention and control group prior the intervention taken place. Counseling only increased husbands’ score from 61.5 to 62.6 (2%), whilst the combination of counseling and *Suami Siaga Plus* application boosted 20% of the husbands’ score from 60.4 to 72.9 (p-value 0.000). The present study also shows the application will gradually improve husbands’ total BP/CR scores. Husbands’ knowledge and practice of birth preparedness and complication readiness were maintained increased until delivery when they following the application’s guidelines. At this point, since the beginning when their wife was in the third trimester of pregnancy until the postpartum period, total BP/CR score increased 11.2±2.0 point after counseling, but the score rose 21.2±6.5 point if the counseling was given together with the application.

***Wives’*** ***BP/CR score description:*** Table 3 shows initial knowledge of female participants on key danger signs was somewhat different. More women in the intervention group (68.4%) understood the key danger signs during pregnancy, slightly higher than their counterpart in control area (52.6%). However, the results noted that more women in control group understood better the key danger signs during labor and postpartum (52.6% and 73.7%, respectively), higher than women in intervention group (47.4% and 52.6%, respectively).

**Table 3:** BP/CR score among women

**Indicators**	**Intervention**		**Control**	
	**Before**	**After**	**Before**	**After**
Knowledge of Key Danger Signs				
During pregnancy	13 (68.4%)	17 (89.5%)	10 (52.6%)	11 (57.9%)
During labor	9 (47.4%)	16 (84.2%)	10 (52.6%)	11 (57.9%)
During postpartum	10 (52.6%)	16 (84.2%)	14 (73.7%)	14 (73.7%)
Five standard elements in BP/CR practices				
Choose an appropriate birth location	15 (78.9%)	18 (94.7%)	15 (78.9%)	15 (78.9%)
Give birth with as killed provider	15 (78.9%)	15 (78.9%)	17 (89.5%)	17 (89.5%)
Mode of transport	18 (94.7%)	19 (100%)	11 (57.9%)	12 (63.2%)
Blood donor in emergency case	8 (42.1%)	9 (47.4%)	8 (42.1%)	8 (42.1%)
Save money	16 (84.2%)	19 (100%)	12 (63.2%)	12 (63.2%)

**Table 4:** The total BP/CR score before and after intervention among the wives

**Total BP/CR Score**		**Group**		**p value**
		**Android App and Counseling**	**Counseling**	
1	Before intervention			0.110
	a. Mean ± SD	70.6 ± 8.5	63.9 ± 7.0	
	b. Min-max	53.5-81.7	52.1-73.2	
2	After intervention: Follow up 1			0.000
	a. Mean ± SD	77.8 ± 8.0	66.8 ± 7.1	
	b. Min-max	63.5-90.1	53.5-77.5	
3	After intervention: Follow up 2			0.003
	a. Mean ± SD	80.1 ± 6.9	72.9 ± 7.0	
	b. Min-max	69-90.5	61.6-88.1	

Unlike on key danger signs, women’s knowledge on five standard elements in BP/CR practices showed varying patterns. The information provided by *Suami Siaga Plus *application helped to improve women’s knowledge in choosing appropriate birth location, modes of transport and savings, but did not show a significant different in giving birth attended by skilled providers and donating bloods in emergency case. It also implies that counseling alone was not sufficient to improve women’s knowledge in BP/CR. The data revealed that more wives understood the five standard elements in BP/CR practices and the danger signs during pregnancy, labor, and postpartum if they are given counseling combined with *Suami Siaga Plus* installation in their husbands’ mobile phone.

Unlike among husbands, initial knowledge of female participants was slightly different. Although insignificant, female’s BP/CR scores in intervention group was higher compared to women in the control group. After their husbands installed *Suami Siaga Plus *application in their mobile for three weeks, women in the intervention group significantly (p-value 0.000) improved their BP/CR score about 10%, from 70.6 ± 8.5 to 77.8 ± 8.0. On the other hand, among women whose their husbands did not installed the application, counseling only improved 5 percent of BP/CR scores, from 63.9 ± 7.0 to 66.8 ± 7.1. At the second follow up, women’s score in BP/CR also continue to increase significantly (p-value 0.003) with a mean score 80.1 ± 6.9, higher than their control counterpart who only showed an average score of 72.9 ± 7.0.

The findings of the present study also suggest at the early measurement, wives’ understand the five standard elements in BP/CR practices and the key danger signs better than their husbands (70.6 ± 8.5 versus 60.4 ± 8.7). However, the total BP/CR score among the husbands who received counseling and installed the application on their mobile phone was higher than the wives (80.1 ± 6.9 versus 81.8 ± 6.2).

## Discussion

Generally, BP/CR scores among husbands and women in all elements increased after the intervention, both for participants in intervention and control group. However, the mean difference of BP/CR scores was higher among husbands who received *Suami Siaga Plus *application, compared to those who received counseling only. This study confirmed that the application installed to husbands’ mobile device contributes a positive benefit in improving birth preparedness and complication readiness of its users. 

There has been a bulk of evidences revealed that three delays are the main factors determining maternal deaths in Indonesia and worldwide (5, 10, 18). The present study not only provides an evidence that media may help in health program dissemination effectively, but also to show that media may raise husbands’ awareness as the closest person to pregnant women. When husbands’ awareness as a key factor increases, three delays will be declined, and in turn, maternal deaths will be decreased (11). 

The findings of the study also indicate a positive sign in social structural changes where male dominance is now replaced by gender equity where couples share responsibilities in the households, including making health-related decision. By providing schedule reminders for husbands in ANC, males will be more involved in the family matters, not only as breadwinner as it has long been rooted in Indonesian culture. *Suami Siaga Plus *application, by providing schedule and information related to breastfeeding also encourages males to be more involved when their wives breastfed their babies. Studies found, husband’s supports may increase the quality and quantity of breast milk produced during lactation period because women feel relaxed (19, 20). When postpartum women have no anxiety and stress, prolactin levels will be increased (21) and breastfeeding outcomes will be improved (22). 

Not only for those who exposed by *Suami Siaga Plus *application, the study also found that husbands’ involvement in counseling might improve the BP/CR score. Ideally, maternal health in the family is a shared responsibility of man and woman as a couple, and both of them have equal role in maternal health. Home-based intervention (family environment) had shown a significant and positive influence on birth preparedness and complication readiness (23). Educating women and their partners yields a greater net impact on maternal health behaviors compared with educating women alone (6, 10, 18, 24).

Moreover, the findings of the study also suggested that the methods of health education had an influence on respondents' awareness of obstetric complications. Media smartphone in this case- has been proven as an effective tool in providing health education to community by enabling the knowledge transfer in an easy and convenient way. With the availability of the information at a fingertip, and with the affordability of mobile internet, mobile phones become an effective channel to deliver health information (14, 25, 26) and able to increase knowledge and practice of birth preparedness and complication readiness. In addition, exposure to BP/CR intervention was associated with a decrease of 28% in the risk of MMR (24).

The present study revealed that combination of technology and counseling yield a better result in increasing BP/CR score compared to counseling only. The respondents in the intervention group received information from more than one channels, whilst the respondents in the control group accepted only regular counseling service. The new media such as smartphone offers greater advantages compared to conventional health education such as face-to-face counseling because beside its anonymity, the information provided by the application enable users to repeat the information they are needed over times. The information from the media then will be cultivated and stored in the users’ cognitive and in turn will change their attitudes and behavior (14). 

However, it should be noted that smartphone users are mostly young people from middle to high income families (27). They are most likely high educated and by their nature have higher curiosity and interest in technology and health compared to other groups. This study has its limitation on the intervention bias because the respondents in the intervention and control groups were residing in the same regency, thus, the contamination of information cannot be avoided. In addition, smartphone limits the health education recipients only to those who can afford it. Whilst for those who cannot afford should rely on the conventional media and traditional methods of delivery. Therefore, any health interventions relied on technology should also consider other methods in message delivery to wider its coverage.

## Conclusion

A combination of counseling and *Suami Siaga Plus* Android Application significantly improves husbands and wives’ score on BP/CR compared to those who received counseling only. The study results could be important information for the Department of Health and health professionals to use android application program of *Suami Siaga Plus*, in particular to the husband whose wife is in pregnancy, childbirth and postpartum periods. The purpose of android application utilization is to increase the birth preparedness and complication readiness that would be able to suppress the three delays, which in turn can reduce the maternal mortality rate.
